# Pigeons: A Novel GUI Software for Analysing and Parsing High Density Heterologous Oligonucleotide Microarray Probe Level Data

**DOI:** 10.3390/microarrays3010001

**Published:** 2014-01-03

**Authors:** Hung-Ming Lai, Sean T. May, Sean Mayes

**Affiliations:** 1 School of Biosciences, University of Nottingham, Sutton Bonington, Loughborough LE12 5RD, UK; E-Mails: hung-ming.lai@kcl.ac.uk (H.-M.L.); sean@arabidopsis.org.uk (S.T.M.); 2 Department of Informatics, King’s College London, Strand, London WC2R 2LS, UK; 3 Nottingham Arabidopsis Stock Centre (NASC), University of Nottingham, Sutton Bonington, Loughborough LE12 5RD, UK; 4 Crops for the Future Research Centre, University of Nottingham Malaysia Campus (UNMC), Jalan Broga, Semenyih 43500, Malaysia

**Keywords:** affymetrix, heterologous microarray, oligonucleotide probe selection, Pigeons, probe pair data analysis, SFPs, Xspecies

## Abstract

Genomic DNA-based probe selection by using high density oligonucleotide arrays has recently been applied to heterologous species (Xspecies). With the advent of this new approach, researchers are able to study the genome and transcriptome of a non-model or an underutilised crop species through current state-of-the-art microarray platforms. However, a software package with a graphical user interface (GUI) to analyse and parse the oligonucleotide probe pair level data is still lacking when an experiment is designed on the basis of this cross species approach. A novel computer program called Pigeons has been developed for customised array data analysis to allow the user to import and analyse Affymetrix GeneChip^®^ probe level data through XSpecies. One can determine empirical boundaries for removing poor probes based on genomic hybridisation of the test species to the Xspecies array, followed by making a species-specific Chip Description File (CDF) file for transcriptomics in the heterologous species, or Pigeons can be used to examine an experimental design to identify potential Single-Feature Polymorphisms (SFPs) at the DNA or RNA level. Pigeons is also focused around visualization and interactive analysis of the datasets. The software with its manual (the current release number version 1.2.1) is freely available at the website of the Nottingham Arabidopsis Stock Centre (NASC).

## 1. Introduction

Microarrays have become a powerful and widely exploited tool when studying the complete gene expression profiles of a multitude of cells and complex tissues in many different organisms. The major technical advance was the hybridisation of reverse transcribed RNA from tissues or cells to either cDNA or oligonucleotides fixed on glass slides or on a nylon membrane [1]. High-density oligonucleotide gene expression arrays have recently been applied to many areas of biomedical research to assess the abundance of mRNA transcripts for many genes at the same time [2]. Affymetrix (Santa Clara, CA, USA) generated GeneChip^®^ arrays and dominated the market of high-density microarray for many years. Although significant quantities of informative, reproducible, and high quality data is generated by the use of a GeneChip^®^ for expression profiling, the Affymetrix chips are only available for a limited number of species of eukaryotes and a small number of model/commercial plant species, including *Arabidopsis thaliana*, barley, rice, maize, tomato, soybean, sugar cane, grape and wheat [3,4].

A genomic DNA-based probe selection technology, known as the Xspecies approach, has been developed to investigate the transcriptomes of heterologous plants and to allow the sensitivity of high-density oligonucleotide microarrays to be applied to species where chips have not yet been designed [3,4]. The approach begins with a genomic DNA/DNA hybridisation, hybridising DNA from species X onto an appropriate Affymetrix GeneChip^®^ of a heterologous species. The next step uses a Script to parse an Affymetrix CDF file of the selected chip. The parser uses the CDF file of the chip and the CEL file of the hybridisation to identify and remove “bad” probe-pairs whose perfect match probe intensities are below a cut-off value defined by the user, eventually making a “new” CDF file for Species X [5]. The new probe–masked file, namely the species X.CDF, can be used for Xspecies transcriptomic analysis of RNA hybridisation. Hammond *et al*. [3] showed that the Xspecies approach had been successfully applied to analyzing the transcriptome of *Brassica oleracea* L. by labeling gDNA from *B. oleracea* and hybridising it to the ATH1-121501 (ATH1) GeneChip^®^ array. The approach with heterologous oligonucleotide microarrays was also utilised to profile and to compare the transcriptional levels of *Thlaspi caerulescens* and *Thlaspi arvense*, both being species where no GeneChip^®^ is available [4]. A further application of this novel approach was to examine the evidence for neutral transcriptome evolution in plants by quantifying more than 18,000 genes transcripts at the level of 14 taxa from the Brassica family [6]. However, the original script parser has a specific limitation in choosing the cut-off - the selection of the value is essentially arbitrary, although a more recent iteration does allow a degree of sub-sampling to suggest thresholds. One method to improve on this approach is to generate many custom CDF files according to different cut-offs, from low to high. Then, a range of good probes pairs and probe-sets with respect to the chosen specific cut-offs are obtained. The researcher, using a spreadsheet, plots these data as background information and uses them to finally decide the optimal value of the cut-off and the corresponding CDF file [7]. The approach is valid but is still human-dependent since people choose the threshold based on their observations and experience when looking at the plot.

Recently, oligonucleotide arrays have been used to recognise allelic variation, the variants being termed single-feature polymorphisms (SFPs). The polymorphism is often detected by a single probe in an oligonucleotide array—the so-called “features”. There is no *a priori* understanding of the DNA nature of the polymorphism, simply that it is a reproducible polymorphism. With this cross-species approach using Affymetrix GeneChip^®^s, researchers have the ability to screen hybridisation datasets for potential SFP markers that exist in minor species. Thus, it is essential to design biological and algorithmic approaches for heterologous oligonucleotide microarray analysis, to help facilitate the genomic investigation of minor plants and animals. Here, we have developed an innovative software package “Pigeons”, abbreviated from “Photographically InteGrated En-suite for the OligoNucleotide Screening”, to work towards a solution to the issues mentioned above. Pigeons allows the user to input and analyse microarray data from the Xspecies microarray approach. This can be DNA hybridisations across species, to determine the empirical boundaries for custom CDF files for Xspecies transcriptomics or to examine an experimental design to identify SFPs at single oligonucleotides within the probe-sets, either at the DNA or RNA level. To allow intuitive interaction and final selection of features of interest, we have also developed a specific visualization interface to facilitate navigation through the hundreds of thousands of Affymetrix oligonucleotides.

## 2. Methods and Algorithms

In this paper, there are three algorithms (automated threshold mapping (ATM), dual fold-change (DFC), probe-wise one-sample statistical test (POST)) presented to fulfill the needs of analysing and parsing the Xspecies microarray data at the probe level. We aim to improve on current Xspecies parser scripts by using several traditional and modern computing techniques including interpolation, projection and clustering [8,9]. Meanwhile, recent microarray gene selection approaches, such as a fold-change (FC) analysis and a variety of statistical tests [10,11,12], have also been extended and modified to address the issue of searching for the single oligonucleotides containing the feature of interest.

The experimental material used for this paper is derived from the underutilized African legume species Bambara groundnut (*Vigna subterranea* (L) Verdc.) which is grown as part of subsistence and small-scale agriculture in many of the sub-Saharan countries of Africa [13,14]. A controlled cross between a genotype derived from a wild non-domesticated landrace (VSSP11; Parent 1; P_1_; “
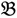
_1_”) and a genotype derived from a domesticated landrace (DipC; Parent 2; P_2_; “
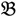
_2_”) was made and a single hybrid seed (F_1_) allowed to grow and produce an F_2_ population of seed. This population was planted and recorded at the Tropical Crops Research Unit at the University of Nottingham in 2003. Individual plants were recorded for numerous traits, including “number of stems per plant”. The extremes of the “number of stems per plant” distribution were identified and 10 plants from each extreme had DNA extracted by standard techniques and mixed in equal amounts to produce a bulked sample of “low stem number” (“
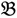
_3_”) and a bulked sample of “high stem number” (“
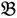
_4_”), respectively.

### 2.1. ATM

**Figure 1:**
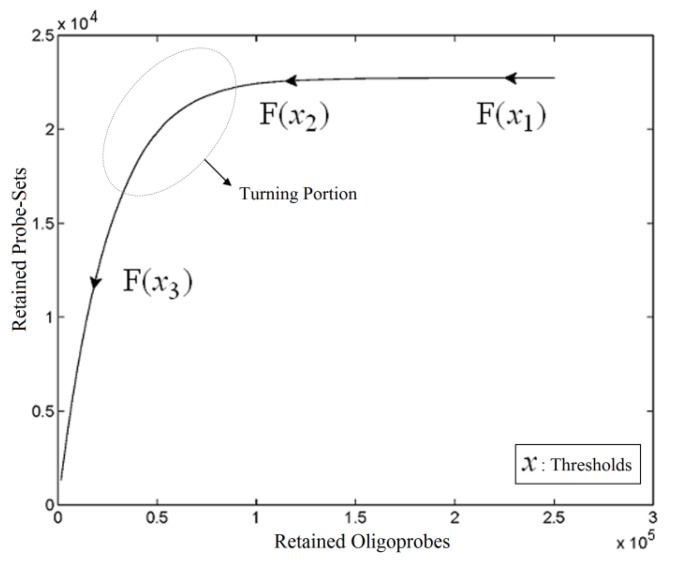
Plane Curve. A vector valued function traced out by retention units with respect to the cut-off of poorly hybridising oligonucleotides using the heterologous GeneChip^®^ platform, with ATH1-121501 used as the basis to generate the image.

As a mixed model of numerical analysis and a soft computing technique, a heuristic method called Automated Threshold Mapping (ATM) was proposed to improve on the human-dependent cut-off selection of poorly hybridising oligonucleotide probes. One of the requirements to be able to exploit an Xspecies array is to select a threshold to generate a custom CDF file for further analysis. Therefore, it is necessary to understand the relationships between a particular threshold value, the probe-pairs and the probe-sets retained at this threshold, *i.e.*, three two-ways and one three-way comparisons. Because this problem involves one input (threshold level) and two outputs (probe-pairs and probe-sets), an idea was drawn from vector calculus to assess the relationships among the three variables and to generalize a solution to this problem. Through the generation of a plane curve (Figure 1), we have found that the retained probe-sets and the retained probe-pairs decline when the threshold value is increased and that the relationship between the two retained variables is a monotonic function. This relates to the point that a probe-set is removed only if there are no retained probe-pairs in that probe-set, so that the number of retained probe-pairs declines more sharply than the number of retained probe-sets does, when the threshold value rises. We also find that the plane curve is like a learning curve with a plateau. Thus, an appropriate selection of threshold values could come from the portion of the curve (circle in Figure 1) between the end of the plateau and the beginning of the linear-like drop. Considering the greyness of the position, we want to provide a suggested threshold value, together with an interval of feasible thresholds available for selection using projection, fuzzy clustering and interpolation techniques. From the observation of the plane curve, given a series of vectors that consist of a threshold and its retained units, the vectors are initially projected onto the retained probe-set space, where fuzzy clustering is performed. Since the section of the curve targeted is a limited bridge between the plateau and the linear-like drop, a good fuzzy clustering approach would lead the bridge to a refined overlap of the first two clusters. Based on this, a suggested threshold value could be produced by an interpolation technique.

To define the methods and principles mathematically, first of all, a vector-valued function is introduced to perform an in-depth analysis of the problem. Let X be a scalar variable and Y be a vector variable with two dimensions. A vector function F: 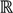 → 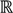
^2^ is defined as follows:

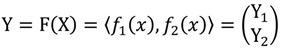
(1)
where X is a set of cut-offs and the component functions *f*_1_ and *f*_2_ are real-valued functions of the parameter *x*. The two components of Y, Y_1_ and Y_2_, are therefore viewed as sets of retained probe-pairs and probe-sets, respectively, when a defined cut-off is given. Using the vector-valued retention function F, we can easily trace the graph of a curve to know the relationships among cut-off and the retention units of probe-pairs and probe-sets. The point of the position vector F(*x*) coincides with the point (*y*_1_, *y*_2_) on the plane curve given by the component equations, as shown in Figure 1. The arrowhead on the curve represents the curve’s orientation by pointing in the direction of increasing values of *x*, namely *x*_3_ > *x*_2_ > *x*_1_. Due to the nature of the problem, the retention function F monotonically decreases in the direction of the point (0, 0). This characteristic means that mapping from *y*_1_ to *y*_2_ is also a monotone function, and moreover, it is actually like a learning curve with a stagnant occurrence.

A tangent vector-based numerical analysis could be applied to the evaluation and the differentiation of the function at a given point. For example, a turning point F(*x_tp_*) can be defined as the intersection between a tangent to the stagnant phase of the curve and the tangent to the linear-like decreasing portion of the curve. The inverse of this point F^−^(F(*x_tp_*)) could be selected as the threshold value. However, the cut-off decision problem is not deterministic, and it usually needs to take biological sense into account, so requires more tolerance in the selection of the threshold. The ATM offers a turning portion (TP) covering the turning point and derived from a closed interval I from which realistic thresholds can be retrieved. Let I be the surrounding area of *x_tp_* ϵ X such that F(*x_tp_*) is the turning point, and then we construct the turning portion by TP={F(*x*): *x* ϵ I }. Construction requires careful definition of a lower boundary (*x_lb_*) and an upper boundary (*x_ub_*) of I, with the aim of developing the idea of selecting a flexible region, rather than a single turning point. Since F is a one-one function well-defined in the interval I, which decreases monotonically; in theory, we can define *x_lb_* and *x_ub_* such that F(*x_lb_*) would be in the terminating phase of the plateau and F(*x_ub_*) would be in the earliest phase of sharp decline, respectively.

The ATM is a data-driven mapping method using a two-stage unsupervised learning process for I determination. The first stage involves orthogonal projection in order to highlight the turning portion. To achieve this, we consider an inner-product vector space 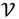 = 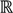
^3^, let 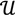 be an *r*-dimensional subspace of 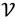 and 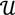
^┴^ be the orthogonal complement of 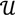. Given a matrix **B***_3_*_×*r*_ such that the column space of **B** is 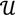, and then for 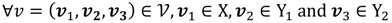 there exists a projector **P** to project *v* onto 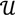 along 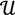
^┴^, *i.e.*, **P***v* = *u*, *u* ϵ 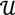. The unique linear operator **P** can be acquired by **P** = **B**(**B**^T^**B**)^-1^**B**^T^, in particular, if **B** constitutes orthonormal bases, then **P** = **BB**^T^. During simplification of the system, the goal at this stage is to minimize the loss of information relevant to the problem of concern. As a consequence, given **B** (e.g., [0,0,1]^T^) and *n* numbers of vectors of thresholds with their retention units, and after linear transformation of each vector *v_i_* ϵ 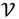, *i* = 1,…, *n* we will then gain a learning data set D = {*u_i_*: **P***v_i_* = *u_i_* ϵ 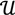 that ideally has the most informative features for turning portion discovery. Suppose that all the data vectors in TP have been projected onto a particular area, we define the area as a hotspot D′ such that

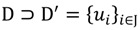
(2)

I = [*x*_inf(J)_, *x*_sup(J)_] (3)

where J is an index set to collect and distinguish the elements of the hotspot, and inf(*J*) & sup(*J*) denote infimum and supremum of J respectively. Obviously, J is a subset of {1, …, *n*}, and both D' and J are well-ordered closed sets.

In other words, the second task is to identify the hotspot to discover a range of feasible cut-off thresholds. Grouping methods would be appropriate for this task as the object of clustering is to group a set of data vectors to include only those vectors which are similar to each other. Although there are similarities between data points within a group to a certain extent, it is also believed that some of the similarities might also occur between groups. This is due to the intrinsic design element aiming to develop a flexible choice of realistic thresholds. Some elements within the turning portion of the curve are closer to the end of plateau, others are near to the beginning of linear-like decline, and still others will be around the turning point of the curve. Part of the problem with depicting the hotspot is to capture the “grayness” of the cross-cluster similarities so it is essential to allow some degree of uncertainty in its description. The ATM applies Fuzzy c-Means (FCM) clustering to this issue since the FCM allows us to build clusters with vague boundaries, where some overlapping clusters include the same object, to a certain degree [15]. Based on an objective function or performance index 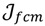, the weighted within-class sum of squares, to quantify how good the quality of clustering models is, the FCM attempts to find the best allocation of data to clusters with a gradual membership matrix **M**. Given a number of clusters *c* (1 < *c* < *n*), then the learning data set 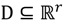 is dominated by fuzzy sets 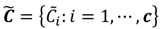 and the fuzzy partition matrix **M** =[*m_ij_*]_*c*×*n*_, where 
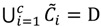
 and 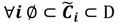. For the individual entries in **M**, *m_ij_* are the membership degree of element *u_j_* ϵ D to cluster *i*, *i.e.*, 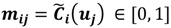. Let 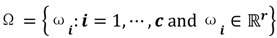 be a set of cluster prototypes so that each cluster 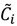 is represented with a cluster centre vector ω_*i*_, and the objective function with two constraints can then be defined as below:

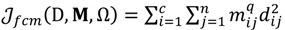
(4)

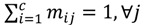
(5)

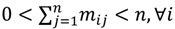
(6)

Here, *q* ϵ 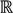
_>1_ is termed the “fuzzifier” or weighting exponent, and *d_ij_* is the distance between object *u_j_* and cluster centre ω_*i*_, within ATM, the Euclidean inner product norm denoted by ‖∙‖ is taken, *i.e.*, *d_ij_* = ‖*u_j_* - *ω_i_*‖. The purpose of the clustering algorithm is to obtain the solution **M** and Ω minimizing the cost function 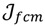, and this can be carried out by:

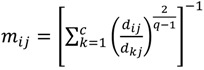
(7)

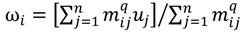
(8)
namely the FCM proceeds with two events: the computation of cluster centroids and the allocation of data elements to these centroids. In practice, the cost function 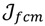 is minimized by an alternating optimization (AO) scheme, *i.e.*, the membership degrees are first optimized given recently fixed cluster parameters, followed by optimizing the cluster prototypes given currently fixed membership degrees. This reiterative procedure will be repeated until the cluster centres have reached equilibrium which is equivalent in mathematics to the optimal objective function 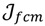.

After the grouping scheme is accomplished, the hotspot D' can be deciphered by defining the greatest lower bound and the least upper bound of its index set. The first two clusters (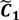, 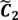) are concentrated for the purpose of deciphering since most of elements of 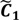 are very likely to be projected from vectors in stagnant phase while data points near the beginning of the sharp drop have mostly fallen into 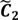. Thus, we let D′ be the subset of the union of the two clusters and set the infimum and the supremum of J according to the objects whose membership values are the maximum of 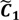 and 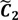, *i.e.*,

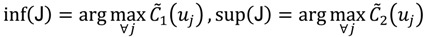
(9)

Not only do the above equations define the index set J, but also they reveal that the tolerance interval **I** has been established. Besides the selection of feasible cut-offs, the ATM also provides an automated threshold value *x_ATM_* and a target interval I' for the selection of candidate cut-offs. Both *x_ATM_* and I' are evaluated through the fuzzy boundary between the first two fuzzy sets. The elements in the boundary imply that 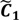 and 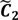 have them in common with various membership values. Owing to the grayness characteristic and the continuity of the learning-like curve, we believe that a good threshold value for parsing the Affymetrix chip description files would come from a projected object that simultaneously belongs to the two clusters with remarkable membership degrees. As a result, the fuzzy boundary can enable us to offer a more reasonable selection of threshold boundary cut-offs. Two indices, *l* and *k*, are utilised to determine the highly likely threshold boundary cut-offs and the automated threshold value, determined by

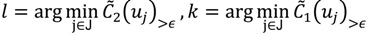
(10)

Here *ϵ* is a small number to assess the possibility of the overlap between the two clusters. By this definition, the fuzzy boundary is then portrayed as the set of 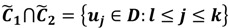 and another closed interval [*x_l_*,*x_k_*] is constructed as the target interval I'. Let be u the arithmetic mean of the elements of 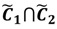, and *x_ATM_* can also be calculated by linear interpolation or by the Lagrange polynomial, as shown in the following formulae:

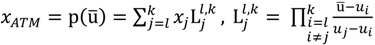
(11)

In summary, the ATM returns a 3-tuple (*x_ATM_*,*I'*,*I*) to resolve the issue of the threshold cut-off choices. The suggested cut-off given by the ATM, *x_ATM_*, can directly be exploited to remove the weak intensity signals while any values within a target interval, I' = [*x_l_*,*x_k_*], can be taken as the potential threshold boundary cut-offs. The design of the target interval gives users a chance of picking a scientifically reasonable value on their own. Those values in a tolerance interval, *i.e.*, *x* ϵ I= [*x*_inf(J)_,*x*_sup(J)_], can be used as feasible thresholds and values outside the interval are viewed as less feasible choices.

### 2.2. DFC

Dual fold-change analysis (DFC) is an approach to seek potential single-feature polymorphism markers through screening all of the 25-mer oligonucleotide probes of the heterologous microarray. Initially, there are two groups (G_1_ and G_2_) under the design of the single trait experiment. While two distinct parental genotype gDNAs are involved in generating G_1_, G_2_ is composed of two different phenotypically based F_2_ bulk segregant pools, derived from a hybrid between the two parental genotypes. We then label the four Xspecies chips with 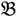
_1_ & 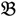
_2_ for the two parent samples and with 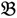
_3_ & 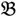
_4_ for the two F_2_ bulks. In practice, these F_2_ bulks are constructed from the pooled DNA of F_2_ individuals. These are derived from the controlled cross between the parental genotypes with allocation to the contrasting bulk based upon a specific trait of interest. The phenotype classification is a necessary prerequisite for the numerical analysis of potential SFP markers. 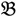
_1_ and 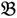
_3_ are classified into one type under a single trait experiment whereas 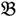
_2_ and 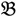
_4_ belong to the other trait version—the prerequisite can be denoted as 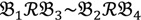. Let N be the number of genes and #(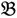) be the cardinal number of a probe-set 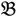 then each chip can be represented as follows:

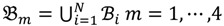
(12)

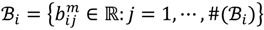
(13)
where *b_ij_^m^* denotes the *j*-th signal intensity of the *i*-th probe-set on the *m*-th chip. Let *Q_ij_^1^* = *b_ij_^1^*/*b_ij_^2^* and *Q_ij_^2^* = *b_ij_^3^*/*b_ij_^4^* be the intensity ratio of G_1_ and G_2_, respectively, thus the ratio value of one for this feature represents unchanged hybridisation signal in this experiment and less than or greater than one is for differentially hybridised oligonucleotides. To generate a symmetric distribution of intensity ratios, the fold-change ratio is defined by

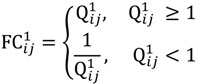
(14)
where FC_*ij*_^1^ is used to assess the differential probe hybridisation of the parental group. For the evaluation of the offspring group, FC_*ij*_^2^ is calculated in the same way as FC_*ij*_^1^ simply replacing Q_*ij*_^1^ with Q_*ij*_^2^. Given the threshold of weak signals *x_ATM_*, the cut-off of a fold-change between the parents 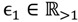 and that between the offspring 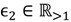, a number of logical criteria are applied to globally screen and search Affymetrix’s single oligoprobes for SFP markers. For 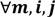, let the first condition be b_*ij*_^m^ > *x_ATM_* since any signals whose intensities are below the threshold should not be used for good probes in the analysis of heterologous data—this satisfies the demand of the XSpecies technology. When the first criterion holds, the DFC enables the procedure to run the second condition with the two fold-change indicators FC_*ij*_^1^ and FC_*ij*_^2^, FC_*ij*_^1^ ≥ ϵ_1_ and FC_*ij*_^2^ ≥ ϵ_2_, to measure whether 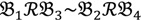 still holds at the genomic level. The FC approach is commonly used in microarray data analysis to identify differentially expressed genes (DEGs) between a treatment and a control. Calculated as the ratio of two conditions/samples, the FC gives the absolute ratio of normalized intensities in a non-log scale. We extend the same concept in our approach by introducing an additional FC—one ratio assesses the differential hybridisation within G_1_ and the other assesses the differential hybridisation within G_2_. The extra FC tests whether the difference in phenotype could result from a difference in genotype at a single locus. Therefore, when there are any differentially hybridised oligonucleotides for the feature of interest between the two parental genotypes, the inherited attribute of 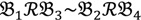 would imply that we could expect those differentially hybridised oligonucleotides to have also been transmitted into the F_2_ individuals. In a word, the corresponding fold-change of the F_2_ is introduced as a cross-check mechanism for identifying SFPs which are consistent between parental genotype/trait and bulk genotype/trait. The mixture of F_2_ genotypes (which are bulked according to the trait difference which segregates within the cross) should mean that the attribute difference is only detected when the location of the parental SFP is close to the gene controlling the trait difference. The accuracy of this approach is dependent upon bulk size used. Smaller bulk sizes will lead to the identification of SFPs which are located distantly from (and probably on different chromosomes to) the target trait associated SFPs. Oligo-probes that satisfy the second criterion above are potential SFP markers distinguishing the two phenotypes and could be further tested and used for genetic mapping of the gene controlling the phenotypic difference.

### 2.3. POST

The FC is typically viewed to be significant if there is at least a two-fold difference [10]. In addition, the FC threshold is selected arbitrarily and does not involve any assessment of statistical confidence so using the FC approach alone may not be optimal [11,16]. Although it is a straightforward and intuitive way to detect oligonucleotides using the dual fold-change criterion, the approach does not engage any evaluation of the significance of differential hybridisation in the presence of biological and experimental variation, which might differ from probe to probe. We have therefore developed inferential statistics herein through a method called the probewise one-sample statistical test (POST) for the assessment of the differential oligoprobe variation observed in terms of statistical power and measures of confidence. We first define an MA-value *ρ_ij_* for the examination of a signal variant in the single trait experiment, for 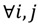 the value is calculated by the following formula:

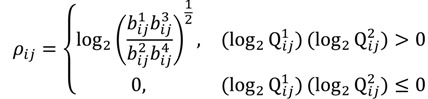
(15)
to exactly correspond with the experimental attribute of 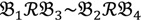. The MA-value is named after the MA plot, a very useful tool in cDNA and GeneChip^®^ microarray data analysis [17,18,19], and is the average intensity ratio between parental samples and F_2_ bulks in a base 2 logarithmic scale with a mnemonic for subtraction and a mnemonic for addition. The POST then uses the MA-value and a single sample *t*-test to statistically assess differentially hybridised oligonucleotides between the parent group and the offspring group and to test in a probe-set *i* whether or not there is significant difference between an interrogated probe *k* and the other probes in that probe-set, in terms of their log ratios. As a test statistic, the average of the MA-values of each of the probe-pairs except the probe *k* is denoted by *ρ_ik_* and determined by:

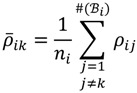
(16)
where *n_i_* = #(*B_i_*) - 1 is the sample size in the examined probe-set *i*. Suppose that the sampling distribution of *ρ_ik_* is normal so that the random variable

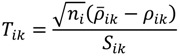
(17)
has a Student’s *t*-distribution with *n_i_* - 1 degrees of freedom. Where *S_ik_* is the standard deviation of the sample of the log ratios in the *i*-th probe-set excluding the MA-value of the oligoprobe *k*. The last step performed by the POST is to asymptotically compute the *p*-value converting the value of *T_ik_* into a probability that expresses how likely the oligonucleotides in question are to be differentially hybridised. To visualize the results of this probewise testing of single oligonucleotides, a filter with a Volcano Plot output was also developed. The volcano plot is an effective and easy-to-interpret scatter plot for the selection of DEGs [11]. In the POST, the plot shows the negative common logarithm (base 10) of the *p*-value *versus* the average intensity ratio in the form of the binary logarithm (base 2), *i.e.*, average fold-change ratio. Probe-pairs with large log ratios and low *p*-values are easily detectable in the view and a list of potential SFP markers can be generated.

Another approach for statistical inference using a different measure based on intensity difference has also been implemented in the POST to identify and evaluate significantly variable oligonucleotides within an experimental group. Basically, the approach is a methodology analogous to that of testing between two groups, but it is more focused on variation within a single group. Since a potential SFP marker could be due to oligonucleotide target regions within the test genome with deletions or duplications or nucleotide differences with respect to the design probe-pairs, we propose using intensity difference rather than the traditional intensity ratio to determine significant differences in intensity between the signal of array elements within either the parent group G_1_ or the offspring group G_2_. We name the intensity difference the *D*-value, in contrast to the MA-value, and define it in compliance with the trait of interest as below:

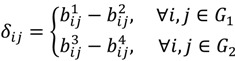
(18)

Similar to statistical tests between groups, the sample mean of the *D*-value would be the statistic to test whether the intensity difference of the oligoprobe under interrogation is significantly different from that of the other signals in the same probe-set of G_1_ or G_2_; meanwhile, an *ad hoc* test procedure within G_1_ or G_2_ also assumes that the population distribution is at least approximately normal and proceeds with the probe-wise strategy. However, there are practical issues that need to be addressed. The majority of intensity signals are likely to be affected by poor hybridisation of the target genome to the heterologous oligonucleotide microarray, leading to the presence of a few or even one possible SFP within a probe-set. The exact number per probe-set will be dependent upon the evolutionary distance between the target species and design array, the rate of evolution of the individual gene represented by the probe-set and the array design itself. Thus, the sample mean is in general a good estimator for the central value of the data distribution of *δ_ij_* when statistical testing is performed according to the probe-wise strategy. But for those probe-sets which have two or more possible SFPs, the mean is no longer an appropriate measure of location under the probe-wise procedure since it will be susceptible to an extreme value. Accordingly, the γ-trimmed mean (0 < γ < 0.5) is employed instead of the mean as the statistic in this version of POST. More mathematically, let 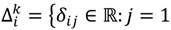, …, *k - 1*, *k + 1*, …, #(*B_i_*} and let *δ_i(1)_* ≤ *δ_i(2)_* ≤… *δ_i(n_i_)_* be the observations of Δ_i_^k^ written in ascending order. We define the sample γ-trimmed mean *δ_ik_* to account for probe-specific fluctuations in a probe-set *i* and its value is calculated by

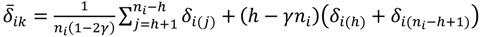
(19)
where *h* = [*γn_i_*] is the value of *γn_i_* rounded down to the nearest integer. Then, let *s_ik_*^2^ be the sample γ-Winsorized variance in the data of Δ_i_^k^ and consider the finite-sample Student-*t* statistic analogue, the γ-trimmed mean can be studentized by *s_ik_* as the form of

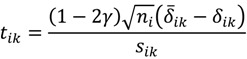
(20)

Tukey and McLaughlin [20] suggested a reasonably accurate approximation of the distribution of *t_ik_* using a Student’s *t*-distribution with *n_i_* - 2*h* - 1 degrees of freedom. Also, Patel *et al*. [21] further introduced a scaled Student-*t* variate *a*(*n_i_*,*h*)*t_ik_* and proposed approximating the distribution of *a*(*n_i_*,*h*)*t_ik_* with a Student’s *t* distribution having *v*(*n_i_*,*h*) degrees of freedom, where *a*(*n_i_*,*h*) = 1 + 16*h*^0.5^*e*^2*h*-*n_i_*^ for small-samples (*n_i_* < 18) *t*-type statistics and *v*(*n_i_*,*h*) has a slight variation depending on γ in their investigation. Given γ = 0.05, 0.10, 0.15, 0.20 or 0.25 we apply the Tukey-McLaughlin suggestion and Patel’s refined approximation to each of *t_ik_* for the calculation of the *p*-value, and the asymptotic *p*-value accompanied with the intensity difference can therefore be prepared for the volcano plot filter and output. To better reveal detection of large-magnitude changes in the output, the POST used the *square-root-transformation* of the *D*-value into the fold-change difference FCD_*ij*_ defined as follows:

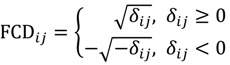
(21)
which produces a symmetric distribution of intensity differences under the assumption that most oligonucleotides are not differentially hybridised, so that the modified volcano plot using fold-change differences is still able to plot changes in both directions, showing equidistance from the centre. Due to the experimental design, the POST tests the inferential statistics on individual oligonucleotides within the parent group and within the offspring group respectively, colouring the plotted points in accordance to the group that they belong to. The colour scheme can be employed as a third dimension of information, for ease of filtering and the setting of parameters. By constructing the coloured volcano plots of G_1_ and G_2_, one can quickly identify the most-meaningful changes in hybridisation signal strength focused on the feature of interest.

## 3. Results and Discussion

### 3.1. Software Implementation

**Figure 2:**
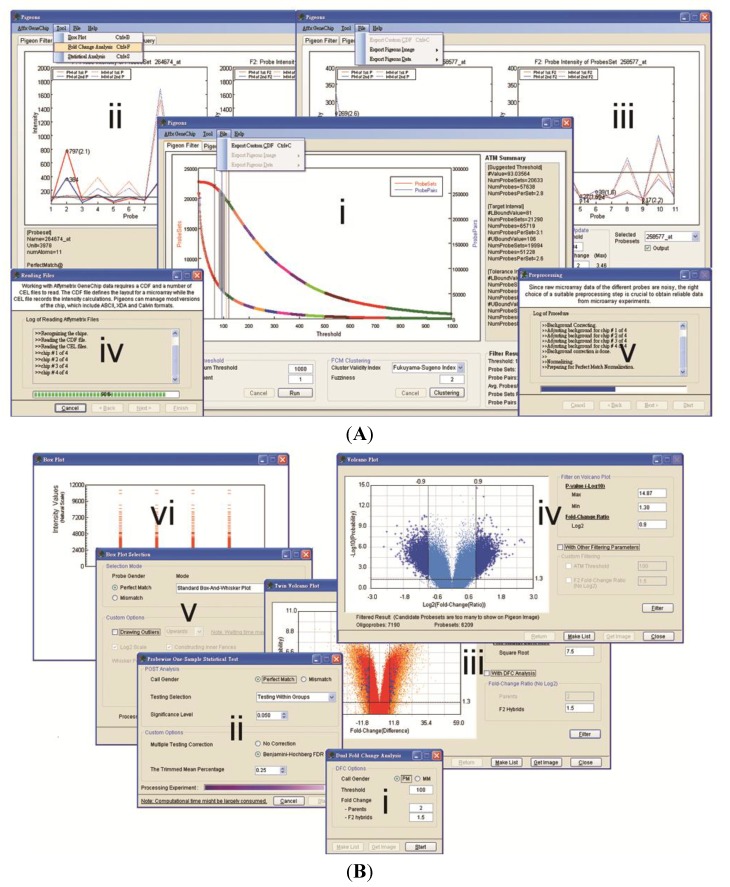
Software Snapshots. Pigeons is a tab-page based standalone graphical user interface (GUI) program. There are three tab-pages for the three main applications in the main form. Each application can be used either separately or jointly. Other tools in a menu strip are also tab-page associated, that is, their availability depends on the application currently being performed. (**A**) Central Applications. The three main applications are: (i) Pigeon Filter; (ii) Pigeon Mining/Image and (iii) Pigeon Query. These are executed after the completion of two core components; (iv) File Reading; and (v) Data Preprocessing; (**B**) Statistical Analyses. Several essential tools can also be called from the menu strip. They are: (i) Dual fold-change (DFC); (ii) Probewise one-sample statistical test (POST); (iii) Twin Volcano Plot; (iv) Volcano Plot; (v) Box Plot dialog-box; and (vi) Box Plot output.

Pigeons is a standalone GUI program for the Windows platform under the .NET framework to analyze Affymetrix GeneChip^®^ data generated from cross-species experiments and the current version number is 1.2.1, released in late-June 2012. The software is able to read most recent or current Affymetrix .CEL file types, including version 3, version 4 and Command Console version 1 (the latest one at the time of program development). It is focused around visualization and interactive studies of data (Figure 2). This computer program is a freeware license so it is free of charge to download and to fully execute for research uses. The .NET Framework version 3.5 or greater is required to install the program. 2 MB of free hard disk space is the minimum to execute the program while 200 MB would be better if data/image file export is required. The golden rule of thumb is that the more RAM the better the capacity, and the faster the microprocessor the quicker the response. At least 1 GB RAM and an Intel^®^ Pentium^®^ M-class processors or better are recommended, although slower CPU speeds with 512 MB system memory will still work in most circumstances. This computer software has successfully been tested on Windows 2000, Windows XP, Windows Vista and Windows 7.

Pigeons is a tab-page-reliant program with the availability of the functions in the main form depending on the tab-page currently presented. There are three tab-pages inside the Windows form. Pigeon Filter is an application to implement the ATM method for the removal of poorly hybridising probe-pairs and to make a probe-masking CDF file (Figure 2(A-i)). Pigeon Mining & Image is developed to perform the DFC analysis approach and the POST statistical filters are used to find potential SFP markers (Figure 2(A-ii,2B-i)). There are two POST-based graphical summary tools within Pigeon Mining (Figure 2(B-ii)). While the Volcano Plot (VP) is used to test differential variation between groups of parents and F_2_ hybrids using the binary average fold-change ratio (Figure 2(B-iv)), the Twin Volcano Plot (TVP) has been designed based on statistical tests within the groups (Figure 2(B-iii)). Results acquired by either the DFC or the POST can be exported as lists and as graphical representations for probe-sets to assist in the interpretation of oligo-level data at the DNA or RNA level. Pigeon Query is an interface for quick probe-set retrieval from datasets (Figure 2(A-iii)). Besides the three main applications, a couple of essential upstream tools are also involved in this software package—they are data preprocessing (Figure 2(A-v)) and a box-and-whisker plot (Figure 2(B-v, 2B-vi)). The Exponential-Normal Convolution Model was utilised for background correction in this program to adjust for systematic effects that arise from variation in the Affymetrix platform [18]. Pigeons employs quantile normalization to address the comparability of intensity distributions between arrays [19]. Then, one can use the box-and-whisker plot, a significant quality control tool, to examine the data before and after data preprocessing. This exploratory data analysis conducts a check for evaluating any extraordinary chip distributions and to verify if a normalization procedure has been effective. A user manual has been provided and built within an installer program so that users can access it from the start menu of MS Windows after the Pigeons has successfully been installed on a local machine. The software with its manual (the current release number version 1.2.1) can be freely downloaded at http://affymetrix.arabidopsis.info/xspecies/pigeons.

### 3.2. Case Studies of ATM

Here, we surveyed a number of previous studies which focused on transcriptome analysis of heterologous species through the across species microarray approach, and compared the cut-off values chosen to make species-specific CDF files in those studies with the ATM’s suggestion based on gDNA hybridisation intensity thresholds (Table 1). *Brassica oleracea* L. (case 1) and *Thlaspi caerulescens* (case 2) were hybridised onto the *Arabidopsis thaliana* ATH1-121501 GeneChip^®^ Arrays [3,4] whereas the two animals (case 4 and 5) were hybridised onto the Human U133 Plus 2.0 Genome Arrays [22,23]. In the third case, the Affymetrix Rice Genome Array was used to investigate transcriptomic profiling related to drought stress in *Musa* [7]. In the original Xspecies approach, *i.e.*, the first case, a probe mask created at a cut-off value of 400 was determined systemically and empirically by generating 13 custom CDF files with a series of gDNA hybridisation intensity thresholds and each CDF was assessed in turn. The probe mask file excluded 68% of the probe-pairs but retained 96% of the available probe-sets, and this was used to study transcriptional response under phosphorus stress. This empirical method of determining the cut-off value was also applied to the second and the fourth cases, which selected the preferred hybridisation intensity thresholds of 300 and of 100, respectively. The same probe selection strategy but subtly different considerations were taken in account in the third and fifth cases. The authors of these two studies determined the hybridisation intensity threshold used to create a probe mask file that was able to detect the maximum possible number of Differentially Expressed Genes (DEGs) even though Hammond *et al*. showed that there was a significant loss of available probe-sets for transcriptomic profiling at the higher end of the cut-off value [3]. As a result, the selected cut-offs used in Banana and Sheep were at the value of 550 and of 450 respectively.

**Table 1:** Summaries of case studies.

Species	Selected Cut-off	Automated Threshold Mapping (ATM)				Reference
		%	Suggested Cut-off	Target Interval	Tolerance Interval	
*Brassica oleracea* L.	400	2.17	391.34 ^a^	[351,426] ^a^	[272,454] ^a^	Hammond *et al*. 2005 [3]
*Thlaspi caerulescens*	300	10.54	331.63 ^a^	[297,363] ^a^	[234,387] ^a^	Hammond *et al*. 2006 [4]
*Musa* (Banana)	550	10.47	492.40 ^b^	[399,586] ^b^	[305,698] ^b^	Davey *et al*. 2009 [7]
Equine (Horse)	100	5.93	94.07 ^a^	[82,106] ^a^	[65,119] ^a^	Graham *et al*. 2010 [22]
Ovine (Sheep)	450	6.93	481.20 ^b^	[381,582] ^b^	[284,694] ^b^	Graham *et al*. 2011 [23]

The cut-off values to mask the intensity signals were examined from 0 to 1,000 with an increment of 1 in all cases. These data sets were then tested under the ATM framework with cluster validation methods to generate the ATM three-tuple result for comparison to the previous publications. % denotes relative difference in cut-off and was calculated from the absolute value of difference between the selected and the suggested cut-off, divided by the selected cut-off value. ^a^ ATM was accompanied by a cluster validation procedure using Fukuyama-Sugeno’s index; ^b^ The partition entropy was applied as a cluster validity index into the ATM algorithm.

Since FCM is an unsupervised process, we introduce two cluster validity indices to accompany the ATM framework to indicate the reliability of clustering results and to cover two different aspects of choosing gDNA hybridisation intensity thresholds. The two cluster validation measures are Fukuyama-Sugeno’s index [24] and partition entropy [15]. In our studies, the first index was exploited in case 1, 2 and 4 whilst the second one was utilised where there was a desire to gain a larger number of differentially regulated transcripts as was the case in 3 and 5; the 3-tuple results of ATM are summarised in Table 1. We found that the hybridisation intensity thresholds selected to understand the transcriptome results in the five cases were all located in the target interval, and they were generally in the vicinity of the cut-off values suggested by ATM. The relative difference in the hybridisation intensity cut-offs were from 2.2% up to 10.5%. Out of the five species, the numerical suggestion of 391.34 by ATM was very close to the biologist’s choice of 400 in *Brassica oleracea* L.—the original research paper presenting the heterologous gDNA hybridisation probe selection approach. The restriction imposed by the researchers of having less than 3% removed probe-sets, led to the selection of an optimal cut-off of 300. This imposed constraint explains the fact that the value of the researcher’s selection was reasonably different to the suggested value given by ATM (331.63) being very near the value of 297, the low end of the target area. ATM was initially developed to find the optimal cut-off of a vector valued retention function (Figure 1) and in practice, the probe mask filter developed using this numerical optimum was able to allow the discover of changes of gene expression in heterologous species. The practical consequence can substantially be shown by the means of the above studies, particularly the first, second and fourth cases. The difference in thresholds between the experienced researcher selection and the ATM’s suggestion in Banana and Sheep was by 6.93% and 10.47%. Not surprisingly, both were higher than those in the other three species, due to the selected stringent criterion for detecting the maximum number of differentially expressed transcripts. By having studied the five non-model plants/animals using model species oligonucleotide arrays, we believe that ATM is valid for the determination of gDNA hybridisation intensity thresholds. The proposed approach can provide fast and objective intensity thresholds, in comparison with the empirical method. When ATM is in operation, we strongly recommend making use of the Fukuyama-Sugeno’s index for transcriptomic and genomics analysis. This index is best for research activities where there is no direct interest in the evaluation of the expressed genes in an experiment, for example, as with finding SFP markers. If the number of DEGs is, however, the major consideration, the partition entropy approach will be a good cluster validity index for this biological purpose.

### 3.3. Examples of an SFP Screen

Besides the generation of an optimal probe mask, a complete solution containing biological and algorithmic approaches to SFP interrogation has been proposed in this article. While DFC is a biology-oriented method and conventionally uses two fold-change with a gDNA hybridisation intensity threshold, POST is a statistically-based and newly-developed procedure with graphical summary filters from two aspects of the test approach.

To evaluate these approaches, we examined bambara groundnut genotypes from an F_2_ offspring derived from a cross between two contrasting parental genotypes. The offspring were bulked according to the trait “number of branches per plant”. Bambara groundnut (*Vigna subterranea* (L.) Verdc.) is an underutilised indigenous African crop species and an important food legume grown widely in sub-Saharan Africa and has been shown to be highly inbreeding. At present, limited sequence resources exist, which means that the Xspecies is a valid approach. The gDNA-based probe-selection using heterologous oligonucleotide microarrays allows us to interrogate thousands of SFPs in parallel and, through the current design, should allow us to efficiently discover markers in a genomic region associated with a specific phenotype. As an illustration of this point, we selected the agronomic trait “number of branches per plant” in a cross between a wild accession with a spreading habit and a cultivated accession with a bunched habit [13,14]. Cross-hybridisation of bambara groundnut DNA from the two parental landrace genotypes VSSP11 (few stem per plant) and DipC (many stem per plant) were conducted using the Affymetrix Arabidopsis ATH1 GeneChip^®^. Meanwhile, two bulks from F_2_ individuals (10 individuals each, representing the high and low stem number extremes from 96 individual F_2_ plants) were hybridised separately onto the Arabidopsis ATH1 GeneChip^®^ array. The experiment was therefore composed of four gDNA hybridisation chips and their relationship could be represented as 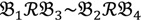, as defined in the methodology section. The probe-level raw data were then background-adjusted and quantile-normalized using the RMA method [18,19] so that these preprocessed intensity signals could be carried over into high level analyses.

**Figure 3:**
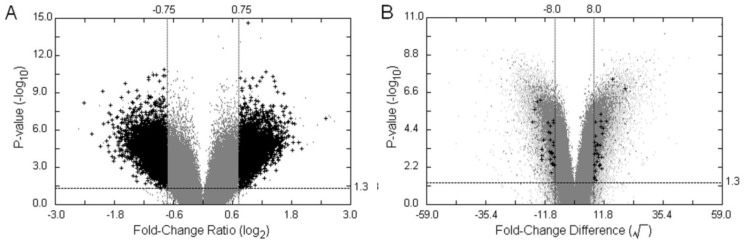
Filtering on Volcano Plots. The customised Volcano-plot tools depicting estimated fold-change (x-axis) and statistical significance (−log10P-value, y-axis) were created by means of the POST inferential statistics for filtering on screening of the single oligonucleotides related to the trait of interest. Each point represents an oligonucleotide probe, and the black crosses corresponded to large fold-changes with a *p*-value less than the significance level or the user-defined value under a number of filtering criteria. (**A**) Volcano Plot (VP). This is an example of applying the POST approach to test between groups of parents and F_2_ hybrid bulks using the binary average fold-change ratio, the MA-value; (**B**) Twin Volcano Plot (TVP). This is an illustration of another version of POST—testing oligonucleotide probes within a parental group and within an offspring group, respectively, followed by plotting the two graphical summaries together in different colours. Light-gray spots were the output of the parental group and gray ones represented the group of F_2_ hybrid bulks. The fold-change difference was defined by transforming the intensity difference D-value into its square root, and was used as a measure to identify the significant intensity differences in the plot.

Figure 3 illustrates two graphical filters, VP and TVP, generated by the POST’s two different visual outputs based on an interrogation of the statistically significant differential hybridisation between the two bulks of bambara groundnut in relation to the trait “stem number”. To correct for multiple testing, we implemented an approach based on controlling False Discovery Rate (FDR), as proposed by Benjamini and Hochberg [25]. The BH adjusted *p*-values were transformed into inverse significances in both VP and TVP, and the suspected SFPs can be filtered and highlighted by the graphical outputs under a number of conditions. Since the samples of the F_2_ offspring act as a cross-checking mechanism in our experimental design, the fold-change of the offspring (FCF_2_) is used as one of the filtering parameters. Additionally, the optimal hybridisation threshold cut-off of the gDNA hybridisation intensity produced by ATM and the cut-off of the parental fold-change used in DFC can be optionally selected to increase the sensitivity of the graphical filters. The 7,903 differentially hybridised signals were summarised (BH adjusted *p* < 0.05, *MA* ≥ 0.75, *MA* ≤ -0.75, FCF_2_ ≥ 1.5) when the POST procedure was performed between the group of parents and of F_2_ samples (Figure 3(A)). The lower levels of hybridisation of features will be more likely to show a significant difference between parental genotypes by chance than high level differences in hybridisation, although the latter could represent repetitive elements within the bambara groundnut genome. Due to the scale of the binary fold-change ratio, this phenomenon is quite common in microarray data analysis. The same preprocessed data set was tested using the other version of POST to examine intensities within groups, followed by filtering potential SFPs using the coloured TVP (Figure 3(B)). Interestingly, there were only 59 probe-pairs (BH adjusted *p* < 0.05, *FCD* ≥ 8, *FCD* ≤ -8, FCF_2_ ≥ 1.5) detected as statistically differentially hybridised using the probewise strategy. The sharply reduced number from thousands to dozens shows that the *D*-value is highly selective against low intensity signals and that the design of TVP, disjointed testing on two groups with a process of filtering in relation to each other, was much more sensitive than the approach of VP based on the average fold-change ratio. 

To have a deeper understanding of the practical effects of using different approaches for SFP detection, various conditions of VP, TVP and DFC were systemically examined and are briefly described in Table 2. Two-fold change is normally the cut-off accepted in microarray analysis. However, the value of 1.5 was adopted rather than 2 for the cut-off of F_2_ in our illustration since the stringent conditions used led to very little in dual fold-change analysis and the hybridisation molecule in this case is genomic DNA, rather than dealing with expression values for RNA. As such, we might expect there to be a similar “dosage” of each gene in the individual genotypes, in the absence of wide-spread duplications. There were four instances inspected using VP and TVP, respectively whereas two cases were considered in DFC. Initial filtering parameters were fixed in the four instances of VP (BH adjusted *p* < 0.05, *MA* ≥ 0.75, *MA* ≤ -0.75) and TVP (10% trimmed mean, BH adjusted *p* < 0.05, *FCD* ≥ 8, *FCD* ≤ -8) and in the two instances of DFC (FCP ≥ 2, FCF_2_ ≥ 1.5). ATM with Fukuyama-Sugeno’s index producing the three-tuple suggestion (93.04, [81,106], [63,120]) of gDNA hybridisation intensity cut-offs for the cases of VP3, 4 and DFC2. Only the perfect match features of the ATH1 GeneChip^®^ was considered in these investigations. When filtering on VP and TVP using initial conditions of x and y axis without extra parameters, we found that VP1 identified more than ten thousand potential SFPs. This was eight times the number using TVP1. This large difference was similar to our findings in Figure 3. We also noticed that the number of differentially hybridised features significantly declined from VP1 to VP2 and very dramatically dropped from VP1 to VP3. These results reveal that the gDNA hybridisation intensity threshold is an essential parameter in the VP filter and low signal hybridised probe-pairs were largely generated in the experiment. This is consistent with the phylogenetic distance between *Vigna subterranea* L and *Arabidopsis thaliana*. When all conditions were applied in VP4 and TVP4, there were approximately equivalent numbers of potential SFPs identified in the two cases, 10 and 8, respectively. An analogous situation between VP1 and VP3 could be found in the investigation of DFC as well. While 3,360 differentially hybridised features were detected in DFC1, very surprisingly, there were just 5 probable SFPs discovered in DFC2—the lowest number out of ten examined conditions. This implies that dual fold-change analysis would be the most stringent approach among the three methods. From the outcomes of VP4, TVP4 and DFC2, where few SFPs were identified we can conclude that the Affymetrix ATH1 GeneChip might not be the best array for heterologous genomic DNA hybridisation with a view to interrogation of the bambara groundnut genome, due to the distant evolutionary relationship between *Arabidopsis thaliana* and bambara groundnut.

**Table 2:** Screening for differentially hybridised oligonucleotides by filtering on two types of volcano plots and dual fold-change analysis under a number of criteria.

Method	Filtering Criteria				Number of potentiallydifferential hybridization ^d^	
VP	*p*-value ^a^	MA-value	FCF_2_	TH ^b^^,c^	Probe-pairs	Probe-Sets
VP1	<0.05	≥|0.75|	-	-	13,694	10,492
VP2	<0.05	≥|0.75|	≥1.5	-	7903	6722
VP3	<0.05	≥|0.75|	-	>93.04	125	124
VP4	<0.05	≥|0.75|	≥1.5	>93.04	10	10
TVP ^e^	*p*-value ^a^	FCD-value	FCF_2_	FCP	Probe-pairs	Probe-Sets
TVP1	<0.05	≥|8.0|	-	-	1,637	1,563
TVP2	<0.05	≥|8.0|	≥1.5	-	59	59
TVP3	<0.05	≥|8.0|	-	>2	50	50
TVP4	<0.05	≥|8.0|	≥1.5	>2	8	8
DFC	FCP	FCF_2_	TH ^b^^,c^		Probe-pairs	Probe-Sets
DFC1	≥2	≥1.5	-		3,360	3,132
DFC2	≥2	≥1.5	>93.04		5	5

The total number of interrogated probe-pairs and probe-sets is 250,103 and 22,746 respectively. Abbreviations. VP: volcano plot; TVP: twin volcano plot; DFC: dual fold-change analysis; FCP: the cut-off of parent fold-change; FCF_2_: the cut-off of F_2_ fold-change; TH: the genomic DNA hybridisation intensity threshold; MA-value: binary average fold-change ratio; FCD-value: fold-change difference as the square-root-transformation of the *D*-value. ^a^ Benjamini-Hochberg adjusted *p*-values were calculated for multiple testing correction; ^b^ The mask of multiple chips was applied. A technique where each signal is extracted from the minimal intensity of four gDNA chips in the single trait experiment to create a pseudo array that will be analysed under the ATM framework; ^c^ Fukuyama-Sugeno’s index was used to generate ATM-suggested gDNA hybridisation intensity threshold; ^d^ SFPs were examined on the Perfect Match probe datasets in all cases; ^e^ 10% trimmed mean, γ = 0.1, of intensity difference was used.

**Figure 4:**
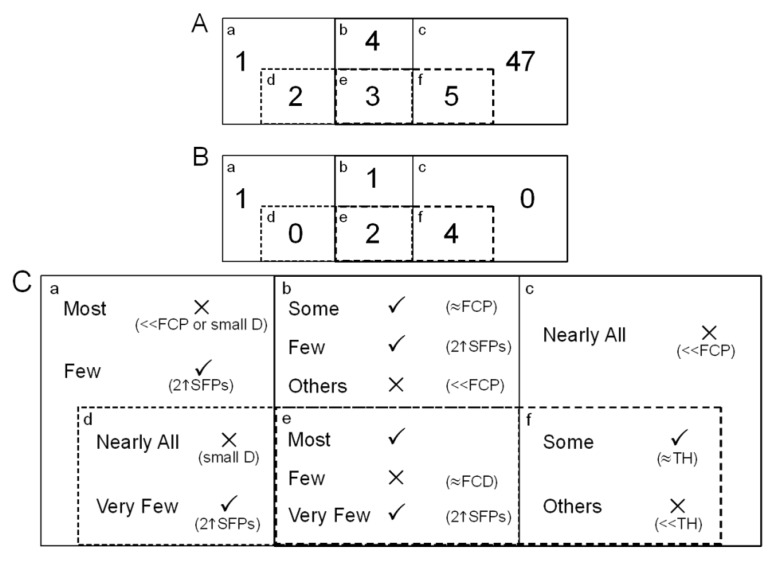
Euler Diagram Analysis. This was an example to show how potential SFPs can be selected by the POST and the DFC using Pigeons. The four-set diagram was established according to VP4 (*abde*), DFC2 (*de*), TVP2 (*bcef*) and TVP4 (*ef*) illustrated in Table 2, where lowercase letters stand for the portions of the four filtering methods. (**A**) SFP Candidates. Numbers in the partitions indicate the number of detected probe-pairs that can be recognised as potential SFPs; (**B**) Final Candidates. After careful selection and consideration portion by portion, potentially differentially hybridised oligonucleotides could be determined. They were *e*:264674_at_473_177, 257321_at_566_65; *b*:258467_at_680_81; *f*:244964_at_665_15, 255530_at_691_371, 257050_at_8_423 and 266293_at_656_319; *a*:265228_s_at_195_89; (**C**) Optimal strategy for potential SFP selection. Where √: candidates; ×: elimination, ≈FCP: the parental fold-change value is just below cut-off; «FCP: the parental fold-change value is significantly below cut-off, small D: little intensity difference; ≈FCD: the fold-change difference value is slightly above cut-off; «TH: poor hybridisation; ≈TH: the signal intensity is a little lower than the value of gDNA hybridisation intensity threshold; 2↑SFPs: there are more than or equal to two potential SFPs found in the same probe-set.

Among the ten instances, VP4, TVP2, TVP4 and DFC2 were selected to acquire more dependable SFPs through Euler diagram analysis. Since TVP2 (*bcef*) and VP4 (*abde*) were proper supersets of TVP4 (*ef*) and DFC2 (*de*) respectively, we can produce a simplified version of the 4 unit diagram (Figure 4). As seen in Table 2, DFC takes advantage of the hard cut-off values of FCP and genomic DNA hybridisation intensity and this approach has a limitation—it may cause possible oligonucleotides to be omitted where they detect repetitive elements within the genome of an investigated species. The set constructed by subtracting DFC2 from VP4 would be able to overcome this potential limitation of DFC. So would the difference between TVP2 and TVP4. The intersection of four units, *e*, is a focus from which the most probable candidates can be found. In the example, 3 suspected probe-pairs were found in this intersection (Figure 4(A)) although one of them was not considered as a potential SFP since its square root intensity difference was not much greater than the FCD cut-off (data not shown). An area, *b*, where the overlap between VP4 and TVP2 but excludes DFC2 is another focus. The elements of this area have potential as their parental fold-changes approach the cut-off value and the signal intensities are not at the low end of the range. To take 258467_at_680_81 as an example, its parental fold-change was 1.96 (564/288), with strong hybridisation and a ratio very near to the cut-off of 2. There were 2 and 5 oligoprobes discovered in the sets of *d* and *f* (Figure 4(A)), respectively, and both *d* and *f* were associated with FCP & FCF_2_. Of the two possibilities for SFPs, the latter seemed more likely. Although the identified oligoprobes exceeded the ATM’s suggested threshold and the cut-off based on the two fold-change parameter, they did not have a particularly large intensity difference (data not shown) so should probably not be selected as candidates. On the other hand the partition *f* has potentially large FCD-values with signal intensities slightly smaller than the gDNA hybridisation intensity threshold based on the ATM suggestion. Out of the 5 filtered entities, there was only one having very poor hybridisation (42 *vs*. 93.04), and this was discarded. The partition built by deducting the intersection of the four units from TVP4 is able to complement another potential constraint of DFC—the hard cut-off value of gDNA hybridisation intensity. When it comes to the area where TVP2 excludes VP4 & TVP4, there were 47 candidates, the largest number in the Euler diagram, detected as statistically significant variable probe-pairs (Figure 4(A)). However, we did not consider any of these as potential SFPs. The reason is that nearly all elements of this set have a much smaller parental fold-change than the given cut-off. Similarly, most discovered probes in the portion where VP4 excludes TVP2 & DFC2 have either small intensity differences or small parental fold-change. In this analysis, there was one probe, 265228_s_at_195_89, belonging to this type of set and we regarded it as a candidate because of its strong hybridisation and reasonable parental ratio of FC (1822/962). The Euler diagram was then updated to show the situation of retained candidates in the units (Figure 4(B)). Eventually, this informed selection enables us to produce a final list of potential SFPs for further validation *in vitro*.

Through this small-size demonstration, an optimal strategy based on the Euler diagram for the selection of differentially hybridised oligonucleotides using POST and DFC has been summarised (Figure 4(C)). Using this strategy, researchers could determine a final candidate list. Firstly, we suggest neglecting the subsets *c* and *d* and picking the elements of the intersection of four-set Euler diagram *e*. Next, the two buffers, *b* and *f*, need to be thoroughly examined as to whether there are any elements whose parental fold-change (for *b*) and signal intensities (for *f*) approximate to the predefined cut-off values, respectively, to find statistically significant variable probe-pairs. Finally, partition *a* should be checked to see if those signals which have strong hybridisation as well as a parental fold-change approaching the cut-off. In addition, there is some opportunity to identify a probe-set having differentially hybridised probe-pairs with more than or equal to a two-fold difference in this partition. Ideally, a probe-set containing multiple SFPs ought to be detected in the intersection of TVP and VP if the trimmed mean percentage γ can be carefully chosen. In our example, γ = 0.1 was used, implying the detection of two SFPs in the same set, and we did not discover any probe-sets with this observable property, arguing against differences between the two parental genotypes (and their offspring) involving the complete absence of probe-sets or their duplication in one genotype only. Making the most of VP, TVP and DFC, the recognition of differentially hybridised oligonucleotides associated with the phenotypic region in a non-model species could be increased.

## 4. Conclusions

Oligonucleotide microarrays have been verified as a powerful high-throughput technology to study plant genomics and transcriptomics. While most arrays are designed for model and major species investigation, there is limited availability of designed microarray platforms for the study of minor crop species that might currently be important food sources in some countries and have potential for future food production more widely. With the advent of the high density oligonucleotide arrays, Xspecies can be used to investigate the transcriptomes of underutilised plants. We have developed several computational algorithms and statistical methods to accompany this oligonucleotide probe-based cross-species platform for the analysis of oligoprobe selection/parsing and for finding potential SFP in minor crop species. These methods have been packaged in a computer program, named Pigeons, focused around visualization and interactive studies of the datasets at the probe level. A number of case studies and an illustration of the analysis of an underutilised crop dataset using Pigeons have also been performed to show the effectiveness and the usefulness of the proposed methods.
